# Keep it simple. A ten-year experience in reconstructions after Mohs micrographic surgery^⋆^^⋆⋆^

**DOI:** 10.1016/j.abd.2020.05.004

**Published:** 2020-09-17

**Authors:** Caroline Martins Brandão, Ellem Tatiani de Souza Weimann, Luiz Roberto Terzian, Carlos D'Apparecida Santos Machado Filho, Francisco Macedo Paschoal, Paulo Ricardo Criado

**Affiliations:** Dermatology Department, Centro Universitário Saúde ABC, Santo André, SP, Brazil

**Keywords:** Carcinoma, basal cell, Dermatologic surgical procedures, Mohs surgery, Surgical flaps, Wound closure techniques

## Abstract

**Background:**

Mohs micrographic surgery is worldwide used for treating skin cancers. After obtaining tumor-free margins, choosing the most appropriate type of closure can be challenging.

**Objectives:**

Our aim was to associate type of surgical reconstructions after Mohs micrographic surgery with the characteristics of the tumors as histological subtype, anatomical localization and especially number of surgical stages to achieve complete excision of the tumour.

**Methods:**

Transversal, retrospective analyses of medical records. Compilation of data such as gender, age, tumor location, histological subtype, number of stages to achieve clear margins and type of repair used.

**Results:**

A total of 975 of facial and extra-facial cases were analyzed. Linear closure was the most common repair by far (39%) and was associated with the smallest number of Mohs micrographic surgery stages. This type of closure was also more common in most histological subtypes and anatomical locations studied. Using Poisson regression model, nose defects presented 39% higher frequency of other closure types than the frequency of primary repairs, when compared to defects in other anatomic sites (*p* < 0.05). Tumors with two or more stages had a 28.6% higher frequency of other closure types than those operated in a single stage (*p* < 0.05).

**Study limitations:**

Retrospective study with limitations in obtaining information from medical records. The choice of closure type can be a personal choice.

**Conclusions:**

Primary closure should not be forgotten especially in surgical defects with fewer stages and in non-aggressive histological subtypes in main anatomic sites where Mohs micrographic surgery is performed.

## Introduction

Mohs micrographic surgery (MMS) is a standard procedure^1^ used for treating skin cancer, especially in cases that fit appropriate use criteria.^2^ In Brazil, this procedure is extensively used.^3^, ^4^

Regardless of the country or dermatologic surgeon’s experience, MMS is always defiant. Its first aim is always to obtain free margins, that lead to higher cure rates than traditional surgical excisions do.^5^ The second aim, is to reconstruct defects with good aesthetic outcomes.

Several types of reconstructions can be used: bi-layered linear repair, flaps, skin grafts, and second intention.^6^ Choice will depend on the type and size of the defect, patient’s characteristics as well as surgeon’s skills and preferences.^7^

In dermatologic surgery literature, a dozen flaps and new surgical approaches and techniques for closing surgical defects are described each year. The choice of type of closure after MMS can be a real challenge, and the “*furor operandi*” described by Max Thorek^8^ as a furious urge to operate and perform huge procedures can sometimes affect Mohs surgeons. Alhaddad et al.^9^ showed that more years of experience was significantly associated with higher uses of flaps and grafts in practice.

In this article, a ten-year experience of an MMS teaching institution was reviewed to describe main techniques used for reconstructions after MMS. The aim was to associate the types of reconstruction selected by surgeons and histological tumor subtypes, its location or number of phases required to obtain free margins.

## Materials and methods

Transversal analyses of medical records of basal cell carcinomas (BCC) that had undergone MMS in our dermatologic surgery unit from May 2005 to May 2015. The study was approved by Ethics Committee (protocol number 1.685.570). Compilation of data such as tumor location (categorized as scalp, forehead, temple, periorbital area, ear, nose, upper and lower lip, cheek, chin, mandible or jaw, neck, chest, back or shoulder, upper extremity, lower extremity, and other), histological subtype (nodular, pigmented, superficial, adenoid, solid, sclerosing/morpheaform, micronodular, basosquamous – if the tumor had more than one histological subtype, the most aggressive one was adopted), number of stages (surgical phases) to achieve tumor clear margins, and type of repair after tumor clearance (bilayered linear repair, advancement flap, rotation flap, transposition flap, island pedicle flap, full-thickness or partial-thickness skin graft, paramedian forehead flap, second intention or referral to plastic surgery or oculoplastic surgery).

### Statistical analysis

Data collected from medical records were deposited in a database, in software Statistical Package for Social Sciences (SPSS) for Windows version 20.0.^10^ After that, descriptive statistics was applied to obtain absolute and relative frequencies of the analyzed variables and the prevalence of investigated outcomes.

In bivariate analysis, association between each independent variable and surgical closure modalities was verified using chi-square test (χ2).

In order to carry out a simple and trustworthy study, all variables were dichotomized. In multivariate analysis, the Poisson regression model was used to analyze independent variables associated with the study outcome (closure type). Prevalence ratio (PR) was also calculated, and possible confounding factors were controlled (adjusted PR). A *p* < 0.05 was considered significant.

## Results

1037 MMS for removing BCC were performed. A total of 975 of these surgeries were analyzed regarding closure type. 62 cases were excluded due to lack of medical records informations. Linear closure was the most common repair by far (39%) and was associated with the smallest number of Mohs stages (1.55 stages on average). Paramedian forehead flaps and skin grafts had the highest mean stages (2.14 and 2.06, respectively) (Table 1).

**Table 1:** Number of stages in 975 Mohs micrographic surgeries (MMS) evaluated and frequency of each closure type.

Closure type	Mean	50th percentile	Range	%
Bi-layered linear repair	1.55	1	1–6	39.2
Advancement flap	1.72	2	1–4	13.8
Rotation flap	1.64	2	1–3	4.8
Transposition flap	1.87	2	1–8	19.7
Island pedicle flap	1.67	2	1–3	1.7
Skin Graft	2.06	2	1–10	10.0
Paramedian forehead flap	2.14	2	2–3	0.7
Second intention	1.86	1	1–7	2.9
Referred for repair	1.33	1	1–2	0.3
Others	2.00	2	1–4	1.0
Not informed	1.74	2	1–4	6.0
**Total**	**1.72**	**2**	**1**–**10**	**100.0**

Bi-layered linear repairs were also more common when histological subtypes were analyzed (Table 2). Additional descriptive statistics also revealed the predominance of this closure type in most of the anatomic sites listed (Table 3).

**Table 2:** Relative frequency of surgical wound closure after Mohs Micrographic Surgery (MMS) in 975 patients, according to BCC (basal cell carcinoma) histological subtypes treated.

Closure type	Nodular BCC	Superficial BCC	Adenoid BCC	Solid BCC	Sclerosing/ morpheaform BCC	Micronodular BCC	Basosquamous	Unclassified BCC
Primary Repair	43.0	52.6	43.5	47.2	28.3	42.4	25.0	38.0
Advancement flap	14.0	13.2	10.9	11.3	14.6	15.2	16.7	10.1
Rotation flap	7.2	2.6	4.3	7.5	3.6	3.8	2.8	3.8
Transposition flap	18.1	15.8	17.4	17.0	23.9	19.7	19.4	17.7
Island pedicle flap	1.8	0.0	2.2	0.9	2.4	1.9	0.0	1.3
Skin Graft	8.6	7.9	10.9	9.4	13.8	8.7	8.3	8.9
Forehead flap	0.9	0.0	0.0	0.9	0.8	0.4	0.0	1.3
Second intention	2.8	2.6	6.5	2.8	2.4	1.5	13.9	2.5
Referred to repair	0.0	0.0	0.0	0.0	0.4	0.8	0.0	0.0
Others	0.5	0.0	0.0	0.9	2.0	0.8	char" char=".">0.0	1.3
Not informed	3.2	5.3	4.3	1.9	7.7	4.9	13.9	15.2
**Total**	**100.0**	**100.0**	**100.0**	**100.0**	**100.0**	**100.0**	**100.0**	**100.0**

**Table 3:** Relative frequency of closure type after Mohs Micrographic Surgery (MMS) in 975 patients, according to anatomic site.

Closure type	Scalp	Forehead	Temple	Orbital	Ears	Nose	Lip	Cheeks	Nasolabial fold	Chin	Jaw	Neck	Chest	Back	Arms	Legs
Primary Repair	33.3	65.6	45.5	40.5	33.8	29.4	30.2	58.0	44.8	60.0	60.0	87.5	66.7	100.0	100.0	0.0
Advancement flap	8.3	14.8	18.2	15.7	9.2	12.7	32.1	10.2	10.3	30.0	20.0	0.0	0.0	0.0	0.0	0.0
Rotation flap	0.0	6.6	3.0	8.5	4.6	4.4	0.0	5.7	6.9	0.0	0.0	0.0	0.0	0.0	0.0	0.0
Transposition flap	33.3	4.9	9.1	11.8	24.6	28.0	15.1	11.4	10.3	0.0	20.0	0.0	0.0	0.0	0.0	0.0
Island pedicle flap	0.0	0.0	0.0	0.7	0.0	1.5	13.2	1.1	6.9	0.0	0.0	0.0	0.0	0.0	0.0	0.0
Skin Graft	16.7	4.9	6.1	8.5	9.2	14.8	0.0	4.5	3.4	0.0	0.0	0.0	33.3	0.0	0.0	0.0
Forehead flap	0.0	0.0	0.0	0.0	0.0	1.5	0.0	0.0	0.0	0.0	0.0	0.0	0.0	0.0	0.0	0.0
Second intention	0.0	1.6	3.0	3.3	12.3	2.5	1.9	1.1	3.4	0.0	0.0	0.0	0.0	0.0	0.0	0.0
Referred out	0.0	0.0	0.0	2.0	0.0	0.0	0.0	0.0	0.0	0.0	0.0	0.0	0.0	0.0	0.0	0.0
Others	8.3	1.6	3.0	0.0	0.0	0.8	1.9	1.1	0.0	0.0	0.0	0.0	0.0	0.0	0.0	0.0
Not informed	0.0	0.0	12.1	9.2	6.2	4.4	5.7	6.8	13.8	10.0	0.0	12.5	0.0	0.0	0.0	100.0
**Total**	**100.0**	**100.0**	**100.0**	**100.0**	**100.0**	**100.0**	**100.0**	**100.0**	**100.0**	**100.0**	**100.0**	**100.0**	**100.0**	**100.0**	**100.0**	**100.0**

BCCs were classified according to their biological behavior as aggressive and non-aggressive, as reported by the National Comprehensive Cancer Network (NCCN).^1^ In Table 4 analysis, sclerodermiform/morpheiform, micronodular, and basosquamous were classified as aggressive subtypes and the remainder as non-aggressive. Primary repair was more common in "non-aggressive" (46.2%) than in "aggressive" (37.5%) cases (*p* = 0.005).

**Table 4:** Association between histological subtype, BCC (basal cell carcinoma) anatomical site, number of surgery stages and patients’ gender and age group with closure types.

Variables	Closure type						
	Bi-layered linear repair		Others		Total		
	n	%	n	%	n	%	*p*
Histological subtype							0.005
Aggressive	191***	37.5	319	62.5	510	100.0	
Non-aggressive	215	46.2	250	53.8	465	100.0	
Anatomic site							0.000
Nasal	141	30.8	317	69.2	458	100.0	
Other location	260	51.3	247	48.7	507	100.0	
Stages							0.000
Single stage	217	50.5	213	49.5	430	100.0	
≥ 2 stages	184	34.5	350	65.5	534	100.0	
Gender							0.957
Female	249	41.7	348	58.3	597	100.0	
Male	157	41.5	221	58.5	378	100.0	
Age group							0.241
< 60 years	107	44.4	134	55.6	241	100.0	
≥ 60 years	290	40.1	433	59.9	723	100.0	
**Total**	**406**	**41.6**	**569**	**58.4**	**975**	**100.0**	**-------**

p, chi-square test (χ2) probability.

Bi-layered linear repair was done in 31% of surgeries performed on the nose although defects outside this location had a higher frequency (51.3%) of this modality of surgical wound closure. This difference was significant (*p* < 0.001).

Linear repairs were more frequent in single-stage surgeries (50.5%) than in cases with 2 or more surgical phases (34.5%). with significant difference (*p* < 0.001). There was no significant difference in gender and age group categories.

Poisson Regression, with and without adjustment of variables, showed that nose surgical defects presented 39.7% higher frequency over other closure types than the frequency of primary repairs, when compared to defects in other anatomic sites (*p* < 0.05). Also, tumors with two or more stages had a 28.6% higher frequency of other closure types (non bi-layered linear repair) when compared to those operated in a single stage (*p* < 0.05). There was no association between surgical repairs and histological subtypes in regression analysis (Table 5).

**Table 5:** Poisson Regression of other repair types after MMS compared to primary closure.

Variables	PR (95%CI)			*p*	Adjusted PR (95%CI)			*p*
	RP	IL	SL		RP	IL	SL	
Histological subtype				0.092				0.221
Aggressive	1.155	0.977	1.366		1.111	0.939	1.315	
Non-aggressive	1				1			
Anatomic Site				0.000				0.000
Nasal	1.412	1.194	1.670		1.397	1.180	1.654	
Other sit	1				1			
Stages				0.001				0.004
≥ 2 stages	1.322	1.114	1.569		1.286	1.082	1.529	
Single Stage	1				1			

PR, prevalence ratio (PR) with no adjustments - bivariate analysis; 95%CI, 95% confidence interval; IL, inferior limit of 95%CI; SL, superior limit of 95%CI; p, observed significance level; Adjusted PR, PR adjusted between variables (including gender and age group) - multiple analysis.

## Discussion

**Figure 1:**
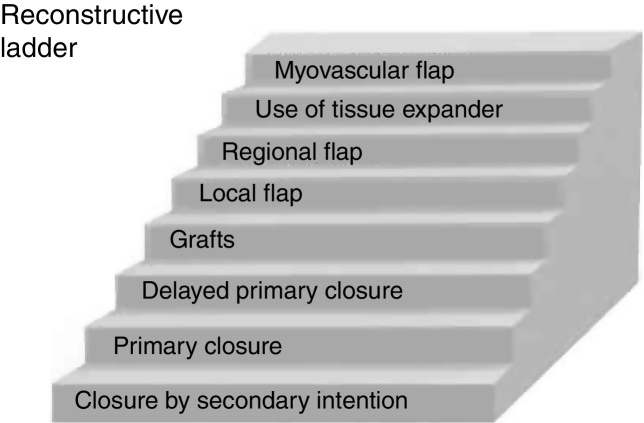
“Reconstructive ladder” demonstrating the simplest to the most complex closures in reconstructive surgery.

The initial goal of this study was to compare different repair types, including several flap modalities, that can be used after MMS, to factors that influenced surgeon’s choice of which type of closure to use. However, after data analysis, overall high frequency of linear repairs was evident. This surgical modality was used in most anatomic sites where MMS was performed. Moreover, it was more common in the tumors with fewer stages and histological subtypes evaluated, in agreement with other similar literature findings.^6^, ^11^ Linear repairs are also one of the first options in reconstructive ladder (Fig. 1) being less complex and having good aesthetic outcomes.^12^, ^13^

An Australian study, evaluating MMS trends after 10 years, showed a decrease in the use of grafts and second intention healing closure types and a significant increase in bi-layered linear repairs utilization.^14^ The increasing tendency to keep reconstructions as simple as possible, since complex reconstructions have higher risks of complications and undesired outcomes, and surgeon’s improvement in skills over the years can justify these findings.^11^

Nose surgical wounds can be challenging even for most experienced dermatological surgeons and it is noteworthy that tumor location can influence the closure type choice^13^ In this study surgical wounds located on the nose showed a 39% chance of being submitted to other closure types rather than to linear repairs when evaluated with Poisson regression model similar to Alam et al.^6^ that also found more flaps than primary closures in nose defects. Transposition flaps can redirect surgical wound tensions and are very useful for defects near free margins such as the nose, eyelids e lip. In our study this surgical approach, despite demanding high complexity in execution and planning, was the second most used flap option among the 975 cases studied demonstrating surgeon’s experiences in reconstructing complex cancer cases and was oftenly used for nose and scalp defects.^15^

Advancement flaps are particularly used for forehead and lip defects to avoid their anatomical distortions. They were frequently used as an alternative option to primary closures in our study is this locations.^16^ It’s well known that aesthetic results of flaps are superior to grafts.^7^, ^17^

Primary Repair was also less common in surgeries that had two or more stages, along with a 28% chance of other closure types in adjusted PR. Simple surgical repairs become more difficult when more stages are needed, in which case, flaps and grafts are required to repair the consequent larger surgical defects.^6^

These other surgical modalities (excluding linear repair) were associated to surgical wounds located on the nose and to aggressive BCC subtypes. These findings alert surgeons for larger surgical challenges when wounds are located some in noble anatomical areas, such as the nose, and when tumors tend to deeply spread.^18^

Bi-layered linear repairs, on the other hand, were more common in fewer stages surgeries, non-aggressive histological subtypes, and lesions located outside the nose. It is not the purpose of this paper to suggest that this closure type should be used for all defects, nor to lessen the importance of flaps. Literature shows that the choice of the closure type depends on defect thickness, patient’s characteristics, and surgeon’s preferences.^7^ However, whenever possible, when proper undermining of all tissue layers can be achieved,^19^ the simplest repair, whether it is linear closure or another alternative with good aesthetic outcomes and that allows better recovery, should be used considering the results shown in this study.

## Conclusions

Reconstruction after MMS can be challenging. During the 10 years of MMS analyzed, primary closures were most used in surgeries with smaller number of phases, non-aggressive histological subtypes and lesions located outside the nose. Tumors at this location and with aggressive histology were associated with the use of other closure modalities. Transposition flaps were the second most used flap and frequently used in nose defects, demonstrating its versatility in this location. Other flaps such as advancement and rotation were used in the labial and peri-orbital regions respectively, making it remarkable that the location of the lesions influences the choice of closure type.

The number of phases may also influence since the use of flaps was more frequent in one third of tumors that required two or more stages for complete resection. The surgeons' passionate desire to perform major procedures described by Max Thorek as “*furor operandi*” may affect Mohs surgeons. However, in surgical defects with fewer stages and in non-aggressive histological subtypes, primary closure needs to be considered as one of the first options in the main anatomic sites where MMS is performed.

## Financial support

None declared.

## Authors’ contributions

Caroline Martins Brandão: Approval of the final version of the manuscript; conception and planning of the study; elaboration and writing of the manuscript; obtaining, analyzing, and interpreting the data; intellectual participation in propaedeutic and/or therapeutic conduct of studied cases; critical review of the literature; critical review of the manuscript.

Ellem Tatiani de Souza Weimann: Statistical analysis; conception and planning of the study; elaboration and writing of the manuscript; obtaining, analyzing, and interpreting the data; intellectual participation in propaedeutic and/or therapeutic conduct of studied cases; critical review of the literature.

Luiz Roberto Terzian: Approval of the final version of the manuscript; conception and planning of the study; intellectual participation in propaedeutic and/or therapeutic conduct of studied cases; critical review of the literature; critical review of the manuscript.

Carlos D'Apparecida Santos Machado Filho: Approval of the final version of the manuscript; conception and planning of the study; obtaining, analyzing, and interpreting the data; effective participation in research orientation; critical review of the manuscript.

Francisco Macedo Paschoal: Statistical analysis, approval of the final version of the manuscript; conception and planning of the study; obtaining, analyzing, and interpreting the data; effective participation in research orientation; intellectual participation in propaedeutic and/or therapeutic conduct of studied cases.

Paulo Ricardo Criado: Elaboration and writing of the manuscript, obtaining, analyzing, and interpreting the data; effective participation in research orientation; critical review of the literature; critical review of the manuscript.

## Conflicts of interest

None declared.
